# Efficient Synthesis of *cis*,*cis*-Muconic Acid by Catechol Oxidation of Ozone in the Presence of a Base

**DOI:** 10.3390/molecules30010201

**Published:** 2025-01-06

**Authors:** Kohtaro Katayama, Hiroki Hotta, Yoshio Tsujino

**Affiliations:** 1 Graduate School of Maritime Sciences, Kobe University, 5-1-1 Fukae-minami, Kobe 658-0022, Hyogo, Japan; 201w801w@gsuite.kobe-u.ac.jp; 2 Graduate School of Science, Technology and Innovation, Kobe University, 1-1, Rokkodai, Kobe 657-0013, Hyogo, Japan; ytsujino@tiger.kobe-u.ac.jp

**Keywords:** *cis*,*cis*-muconic acid, ozonolysis, green chemistry, catechol, synthesis efficiency

## Abstract

Muconic acid, a crucial precursor in synthesizing materials like PET bottles and nylon, is pivotal for the anticipated growth in the textiles and plastics industries. This study presents a novel chemical synthesis route for *cis*,*cis*-muconic acid (ccMA) using catechol. Biochemical methods face scale-up challenges due to microorganism sensitivity and complex extraction processes, while chemical methods involve environmentally harmful substances and have low yields. Our research introduces a method that enhances ccMA yield to 56% by employing ozonation in the presence of an alkali, significantly simplifying the synthesis process. This one-step synthesis reduces reagent use and labor, aligns with green chemistry principles, and avoids using toxic chemicals. The methodology, involving the low-temperature ozonation of catechol with base addition, reduces ccMA degradation and improves yield, as confirmed by an HPLC analysis and replicated experiments. This promising approach could lead to sustainable industrial synthesis of muconic acid derivatives. Further investigations will focus on refining this method for larger-scale applications and testing its economic viability, aiming to optimize conditions for maximum efficiency and yield.

## 1. Introduction

Muconic acid is a natural organic acid that can be used as a synthetic precursor for various raw materials, such as terephthalic acid, used in the production of PET bottles and polyethylene [1], adipic acid, used for nylon-6,6 [2], and ε-caprolactam for nylon 6 production [3,4,5,6,7]. Due to the expected growth in demand for textiles and plastic products, the market size of muconic acid is projected to increase significantly. According to Reference [8], the market is anticipated to rise from USD 102.32 million in 2023 to USD 183.77 million by 2030, at a CAGR of 8.72%. Meanwhile, Reference [9] estimates a growth from USD 111.27 million in 2023 to USD 179.96 million by 2030, reflecting a CAGR of 7.11%. Despite slight variations in these forecasts, both analyses indicate a robust and steady expansion of the muconic acid market over the coming years. A low-cost process for producing muconic acid for commercial use is currently being investigated but has not yet been established [10]. The biological synthesis of *cis*,*cis*-muconic acid (ccMA) by metabolizing natural raw materials, such as glucose, xylose [11,12], and lignin [13,14], by modified E. coli has also been reported. These biochemical syntheses are considered a sustainable approach. However, there are some problems in applying biochemical synthesis to industrial large-scale production, such as the need to use low-concentration culture media to prevent toxicity to microorganisms, and the difficulty of the extraction and purification process of the ccMA produced [15]. Therefore, chemical methods are advantageous for the industrial synthesis of muconic acid.

**Scheme 1:**
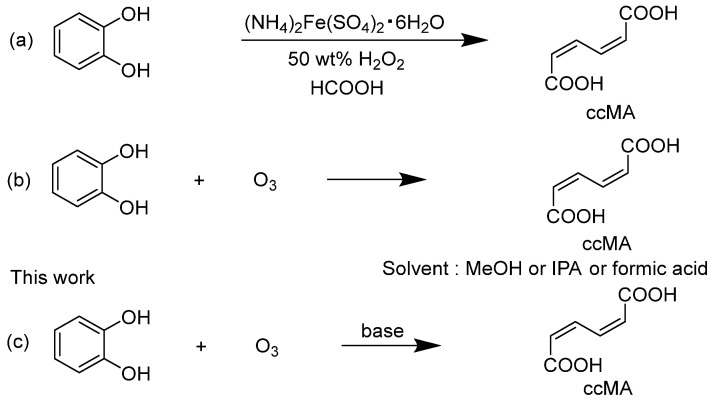
(**a**) Synthesis of *cis*,*cis*-muconic acid (ccMA) by oxidative cleavage of catechol with peracids [15], (**b**) synthesis of ccMA from catechol oxidation by ozone [23], and (**c**) synthesis of ccMA from catechol oxidation by ozone in the presence of an alkali.

A *trans*,*trans*-muconic acid (ttMA) can also serve as an important petroleum-derived chemical feedstock, similar to ccMA. A synthetic method for ttMA has been reported by Guha et al. [16]. But, this process is undesirable from a green chemistry perspective because it is multi-step, in addition to concerns about environmental toxicity and safety hazards. Moreover, the multi-step nature of the process makes it undesirable from a green chemistry perspective. On the other hand, there have been several reports on the synthesis of ccMA by a ring-opening reaction of catechol as a starting material [15]. The reaction in a 50% hydrogen peroxide and formic acid mixture in a solvent yields ccMA at a high yield of about 80% (Scheme 1a). However, this method is not widely practical because of the difficulty of obtaining 50% hydrogen peroxide and the risk of explosive decomposition or chemical reaction explosions at ambient temperature and pressure, which pose significant storage, transport, and handling concerns [17]. In addition, chemical synthesis methods for ccMA and its derivatives have also been reported [18,19]. Since these methods used metal catalysts, post-treatment was complicated for industrial synthesis, and the use of dichloromethane and the large amount of pyridine required were of concern.

In general, the oxidative decomposition reaction of double bonds with ozone is a clean and effective option because it is easy to perform [20]. In two patents by Siggel and Spengler, it was claimed that the yield of ccMA based on the ozonolysis of catechol (Scheme 1b) was between 28.2% and 54.9% [21,22]. But, the actual yields reported in the academic paper ranged from only 0.9% to 31.5%, much lower than the values claimed in the patents [23]. This discrepancy suggests that the reaction conditions described in the patents may not reliably achieve the claimed yields. The reason for the low yield in the reaction with ozone may be that the ccMA produced is decomposed to glyoxylate, oxalic acid, and other acids by excessive reaction with ozone [24,25].

In contrast, we have developed in this study a new method for synthesizing ccMA disodium salt with a high yield of 56% by employing the approach of adding an alkali to the reaction solution (Scheme 1c). This method enabled an efficient one-step synthesis of ccMA from catechol, which significantly reduced the amount of reagents, time, and labor compared to conventional methods. This new approach offers significant advantages, not only from an economic perspective but also in terms of sustainable manufacturing, aligning with the principles of green chemistry.

## 2. Results and Discussion

### 2.1. Decomposition of ccMA by Ozone

**Scheme 2:**
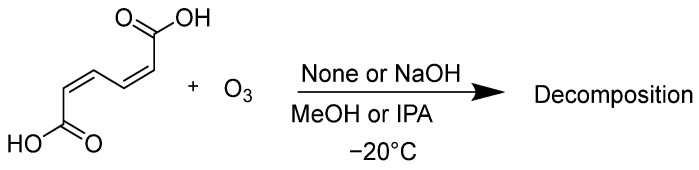
Reaction of ccMA with ozone in alcohol.

At first, the oxidative decomposition of ccMA by ozone was evaluated. The ccMA was dissolved in 2-propanol (IPA) or methanol (MeOH), and oxygen gas containing ozone was bubbled through the solution, either with or without the addition of granulated NaOH (its particle size was less than 0.7 mm) (Scheme 2).

It has been reported that ozone does not react significantly with MeOH when the ozone-reactive olefin is dissolved in MeOH at −20 °C [26]. The data are shown in Table 1.

From the results of entries 1 and 3, the amount of ccMA remaining in the IPA solution after 1 h of reaction was greater than that in the MeOH solution, meaning that the oxidation of ccMA by ozone occurred more slowly in IPA because the solubility of ozone is lower in IPA than in MeOH. In contrast, the addition of NaOH significantly increased the percentage of ccMA remaining for both solvents. In this case, there was no difference in the amount of ccMA remaining in the IPA and MeOH (compare entries 2 with 4). A white suspension was observed in the reaction flask when NaOH was added. These results suggest that the addition of NaOH during the synthesis of ccMA may create a suspension of the sodium muconate ion pair in the solution during the reaction, making it less likely to react with ozone.

### 2.2. Decomposition Rate of Catechol by Ozone and Formation Rate of ccMA

**Scheme 3:**
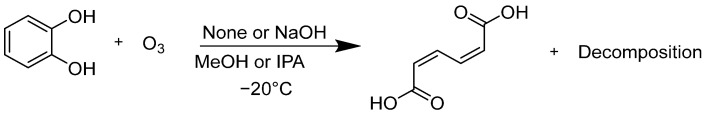
Reaction of muconic acid in alcohol with ozone.

Next, the decomposition rate of catechol and the production rate of ccMA were evaluated (Scheme 3). Catechol was dissolved in IPA or MeOH at −20 °C, with or without NaOH, and ozone gas was bubbled into the solution.

The results are listed in Table 2. Along with the differences in the solvent, differences due to the addition of granulated NaOH were also compared. The catechol concentration (%) is shown as a ratio to the starting concentration, while the ccMA percentage was determined as a ratio of the maximum ccMA production assumed from the initial moles of catechol. When comparing entries 1 and 3 (or 2 and 4), the decomposition rate of catechol was faster in MeOH than in IPA. The slower production rate of ccMA relative to the decomposition rate of catechol may be due to further oxidation of the ccMA by ozone. Comparing entries 1 and 2 (or 3 and 4) showed that the addition of NaOH leads to a more rapid decomposition of catechol. There are two possible reasons for this. One, the redox potential of catechol shifts to the negative side in an alkaline solution, making it easily oxidized. Another is that the chemical equilibrium was biased more toward the product side because the reaction products precipitated as an ion pair, disodium muconate.

The addition of NaOH increased the ratio of ccMA to the amount of catechol decomposed. In this case, NaOH was added three times the moles of catechol. Therefore, the NaOH did not completely dissolve at the beginning of the reaction, but it gradually dissolved as ccMA was produced. When IPA was used as a solvent, the decomposition rate of the catechol was slower, but the conversion ratio to ccMA was higher. This was consistent with reports that muconic acid was formed at a faster rate in low dielectric constant solvents [23]. It was believed that the sodium salt of the ccMA precipitate was more likely to form in IPA, which is more hydrophobic. Thus, an undesirable oxidation of ccMA by ozone was prevented, and the oxidation of catechol proceeded more effectively.

### 2.3. Optimization of Reaction Conditions for ccMA Synthesis

**Scheme 4:**
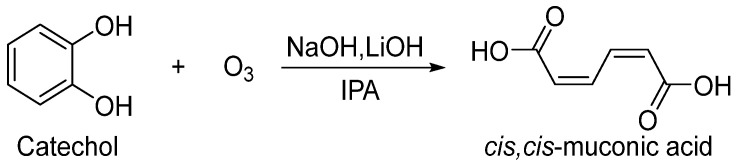
Preparation of ccMA.

The addition of an alkali (such as NaOH or LiOH) to precipitate ccMA as an ion pair was found to be effective in preventing excessive oxidation of ccMA and improving yield. It was also found that IPA was more suitable for precipitation. Therefore, the type of base and the ozone concentration in the reaction solution were further examined (Scheme 4).

Production efficiencies of ccMA under each condition are shown in Table 3. Measurements were taken every 3 h until the catechol disappeared. From entries 1–6, there was no change in yield for less than 6 h, but the yield increased at lower temperatures after 9 h. By decreasing the temperature to −40 °C, the ozone concentration was increased, but the reaction rate slowed down. So, the decomposition of catechol was probably at the same level. The solubility of the disodium muconate was further decreased, suggesting that the ccMA was efficiently synthesized. Experiments using granular LiOH (particle size less than 0.7 mm) instead of NaOH were expected to yield higher efficiency due to the lower solubility of Li^+^ ion pair formation, but the results were obtained with almost the same efficiency (Entry 7–9).

In addition, reactivity was examined when the flow rate of ozone was increased to 50 mg L^−1^, in order to increase the ozone concentration in the solution. From entries 10 to 12, increasing the ozone concentration to 50 mg L^−1^ increased the rate of ccMA formation over a reaction time of 3 h, yielding 56% ccMA in 4.5 h (entry 11). This was the highest yield in this study, and repeated experiments confirmed its reproducibility. The yield was decreased when the reaction time was lengthened to 6 h. This might be due to the decomposition of the suspension of disodium muconate being accelerated by the high concentration of ozone.

When the reaction was performed under the same conditions as in entry 11, but with 1-butanol as a solvent, the yield of ccMA was low, at only 4.7%. This may be due to the low solubility of NaOH in 1-butanol, which made it difficult for the ionic pairs of ccMA to be suspended. The product was obtained as a sodium salt of ccMA, but the acid form was easily obtained by precipitation with an acid in an aqueous solution. A ccMA in acid form was crystallized according to the literature [27], and the ^1^H NMR spectrum was measured (see Supplementary Materials, pages S7 and S8).

## 3. Materials and Methods

### 3.1. Chemicals and Reagents

Catechol (special grade, ≥99.0% purity), methanol (special grade, ≥99.8% purity), 2-propanol (IPA, ≥99.7% purity), NaOH (flake, special grade, ≥97.0% purity), NaOH (granular, 1st grade, ≥93.0% purity), lithium hydroxide monohydrate (special grade, 98.0–102.0%), formic acid (LC/MS grade, ≥99.5% purity), and acetonitrile (HPLC grade, ≥99.9% purity) were purchased from Fujifilm Wako Chemicals (Tokyo, Japan) and used as received. The *cis*,*cis*-muconic acid (ccMA, ≥97.0% purity) was obtained from Sigma-Aldrich (St. Louis, MO, USA). Ultrapure water was obtained from a Millipore MilliQ system (Merck, Darmstadt, Germany).

### 3.2. Apparatus and General Procedures

The apparatus consisted of a 300 mL brown round-bottomed flask placed in a low-temperature thermostatic bath equipped with a magnetic stirrer (details in Supplementary Materials, page S2). The ozone generator (LOG-LC15G, Eco Design Corporation, Saitama, Japan) was used to produce an oxygen flow containing 27–50 mg L^−1^ ozone (see Supplementary Materials, page S3). After the reaction, the solution was analyzed by an HPLC system (Alliance 2695 Separations Module with PDA detector, Waters Corporation, Milford, MA, USA) based on an absorbance at 257 nm (*ε* = 17,300 for ccMA [28]) (see Supplementary Materials, page S4). An ODS column (Inert Sustain C18, 5 μm, GL Sciences, Tokyo, Japan) was used (see Supplementary Materials, pages S5 and S6). The column temperature was set to 30 °C, the flow rate to 1.0 mL min^−1^, and a gradient mobile phase of water/acetonitrile each containing 0.1% formic acid (gradient details were described in Supplementary Materials, page S4). Reaction yields were determined by measuring the ccMA concentration and assuming a 1:1 molar ratio of the formation from catechol.

### 3.3. Evaluation of ccMA Decomposition by Ozone

A commercially available ccMA, weighing 1.4 g (10 mmol), was added to a 300 mL brown-colored flask, and 100 mL of IPA (or MeOH) were added and dissolved. A 1.2 g amount (30 mmol) of NaOH (granulated, particle size <0.7 mm) was added to the flask and stirred at room temperature for 10 min. The flask was placed in a cooling bath at −20 °C and stirred for 10 min. Oxygen containing 27 mg L^−1^ of ozone was bubbled at 1 L min^−1^ for 15 min under magnetic stirring. After that, the ozone generator was turned off, and bubbling with oxygen continued for 5 min at room temperature to remove the residual ozone. Distilled water was added to the reaction solution to dissolve the product precipitate and the undissolved NaOH. The solution was diluted to 1 L in a measuring flask. This diluted solution was further diluted 10-fold, and the solution was analyzed by HPLC as described above.

### 3.4. Determination of the Amount of Catechol Degradation and ccMA Formation by Ozone

A 1.1 g (10 mmol) amount of catechol was added to a brown-colored flask, and 100 mL of IPA (or MeOH) were added, dissolving the catechol. Either 1.2 g (30 mmol) of NaOH was added to the flask, or it was omitted, and the mixture was stirred at room temperature for 10 min. The flask was then placed in a cooling bath at −20 °C and stirred for an additional 10 min. Oxygen gas containing 27 mg L^−1^ of ozone was bubbled into the flask at a rate of 1 L min^−1^. After 15 min, the ozone generator was turned off, and the subsequent treatment was carried out in the same manner as in Section 3.3.

### 3.5. Optimization Study of ccMA Synthesis Conditions

An 11 g (0.1 mol) amount of catechol was dissolved in 150 mL of IPA in a brown round-bottomed flask under stirring. Then, 12 g (0.3 mol) of NaOH or 13 g (0.3 mol) of LiOH·H_2_O were added to the flask. The flask was stirred for 10 min at room temperature under light-shielded conditions, and then, the solution was cooled by setting the flask in a low-temperature thermostatic bath and stirred for an additional 10 min. Oxygen containing 27 mg L^−1^ of ozone was bubbled into the flask at a rate of 1 L min^−1^. After each reaction time, the ozone generator was turned off, and the subsequent treatment was carried out in the same manner as in Section 3.3. The disodium salt was converted to the acid form by adding acid to the aqueous solution and crystallized [28]. The product was identified as ccMA by ^1^H NMR (see Supplementary Materials, pages S7 and S8).

## 4. Conclusions

In summary, we have discovered a method to suppress the decomposition of ccMA produced in the catechol oxidation by ozone and, as a result, successfully increased the yield from the conventional about 30% to more than 50%. It was found that the addition of an alkali increases the rate of reaction between the catechol and ozone and produces a suspension of the reaction product. This prevents the excessive decomposition of ccMA. The yield of ccMA depended on reaction conditions such as solvent, reaction temperature, and ozone concentration. Furthermore, the ability to suppress excessive oxidation and utilize milder conditions without resorting to hazardous reagents, such as peracids, offers clear advantages for large-scale production, potentially reducing operational hazards and environmental impact. Such improvements could pave the way for implementing this method in industrial settings, supporting the broader adoption of green chemistry practices. Further research on improving reaction conditions for higher efficiency and industrial-scale production methods is currently under investigation.

## Figures and Tables

**Table 1:** Decomposition of ccMA by ozone.

Entry	Base	Temp./°C	Solvent	Time/h	ccMA Remaining (%)
1	None	−20	IPA	1	36
2	NaOH	−20	IPA	1	92
3	None	−20	MeOH	1	2
4	NaOH	−20	MeOH	1	92

**Table 2:** Decomposition rate of catechol by ozone and formation rate of ccMA.

Entry	Base	Temp./°C	Solvent	Time/min	Catechol (%)	ccMA (%)
1	None	−20	IPA	15	73	0.38
2	NaOH	−20	IPA	15	26	15
3	None	−20	MeOH	15	59	0.73
4	NaOH	−20	MeOH	15	5.7	15

**Table 3:** Reaction yields of catechol oxidation by ozone in IPA at different reaction temperatures in the presence of various alkalis.

Entry	Base	Temp./°C	Ozone Conc./mg L^−1^	Time/h	Yield (%)
1	NaOH	−20	27	3	24
2	NaOH	−20	27	6	26
3	NaOH	−20	27	9	22
4	NaOH	−40	27	3	23
5	NaOH	−40	27	6	28
6	NaOH	−40	27	9	37
7	LiOH	−40	27	3	24
8	LiOH	−40	27	6	31
9	LiOH	−40	27	9	37
10	NaOH	−40	50	3	25
11	NaOH	−40	50	4.5	56 ^a^
12	NaOH	−40	50	6	33

^a^ The average yield was 50% after three repeated experiments.

## Data Availability

Data are contained within the article and Supplementary Materials.

## Supplementary Materials

The following supporting information can be downloaded at: https://www.mdpi.com/article/10.3390/molecules30010201/s1, Figure S1: Schematic diagram of the synthesis apparatus; Figure S2: Diagram of the ozone generator; Figure S3: Relationship between the dial setting and ozone concentration; Figure S4: Calibration curve for ccMA; Figure S5: Chromatogram after reaction under the conditions of Entry 11 in Table 3; Figure S6: Chromatogram of ozone oxidation of catechol in IPA containing NaOH at 15 min (Entry 2 in Table 2); Figure S7: Chromatogram of ozone oxidation of ccMA in IPA containing NaOH at 1 h (Entry 2 in Table 1); Figure S8: Crystal of ccMA; Figure S9: ^1^H NMR of ccMA; Table S1: Conditions for HPLC measurements; Table S2: ccMA concentration and peak area.
