# Supplementary material for: Efficient Synthesis of cis,cis-Muconic Acid by Catechol Oxidation of Ozone in the Presence of a Base
Source: Molecules. 2025 Jan 6;30(1):201. doi: 10.3390/molecules30010201 (PMC11721871; doi:10.3390/molecules30010201)
Supplement: Supplementary file 1 [file molecules-30-00201-s001.zip › molecules-3386533-supplementary.pdf]

Supplementary

Efficient Synthesis of *cis,cis*-Muconic Acid by Catechol Oxidation of Ozone in the Presence of a Base

Kohtaro Katayama[1], Hiroki Hotta[1,2],* and Yoshio Tsujino[2]

[1] Graduate School of Maritime Sciences, Kobe University, 5-1-1 Fukae-minamimachi, Higashinada, Kobe, Hyogo, Japan.

[2] Graduate School of Science, Technology and Innovation, Kobe University, 1-1, Rokkodai-cho, Nada, Kobe, Hyogo, Japan.

Contents:

1. Experimental details Page **S2**

2. Ozone gas concentration Page **S3**

3. Analytical HPLC chromatograms Page **S4**

4. Conditions for HPLC Measurements Page **S5**

5. Chromatograms Page **S6**

6. Crystallization of ccMA Page **S7**

7. NMR spectra Page **S8**

1. Experimental details

1.1 Apparatus

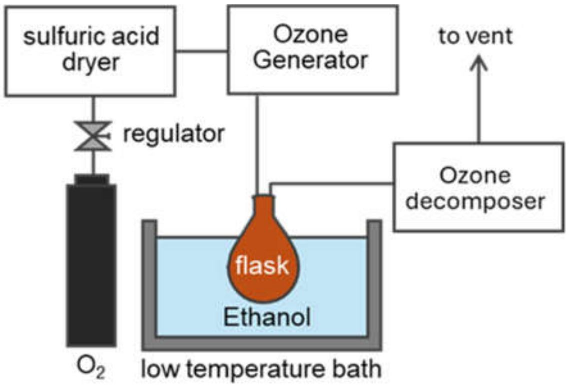

Figure S1 Schematic diagram of synthesis apparatus

The schematic diagram of the synthesis apparatus is shown in Figure 1. The apparatus consisted of a 300 mL brown flask set in a low-temperature thermostatic bath with a magnetic stirrer (PSL-1400, Eyela, Tokyo, Japan), an ozone generator LOG-LC15G manufactured by Eco Design Corporation (Saitama, Japan,) and an ozone decomposer ED-MD9-500S (Eco Design Corporation).

Corrosion-resistant materials were used for the equipment and piping that came into contact with ozone gas. Oxygen gas supplied from an oxygen cylinder was dried with concentrated sulfuric acid and then sent to the ozone generator. The oxygen gas containing ozone was bubbled into a reaction. Unreacted ozone was decomposed in an ozone decomposer and then vented.

Ozone concentration was measured using an ozone gas concentration meter O3M-S-G-300G-P (Eco Design Corporation, Figure S2).

2. Ozone gas concentration

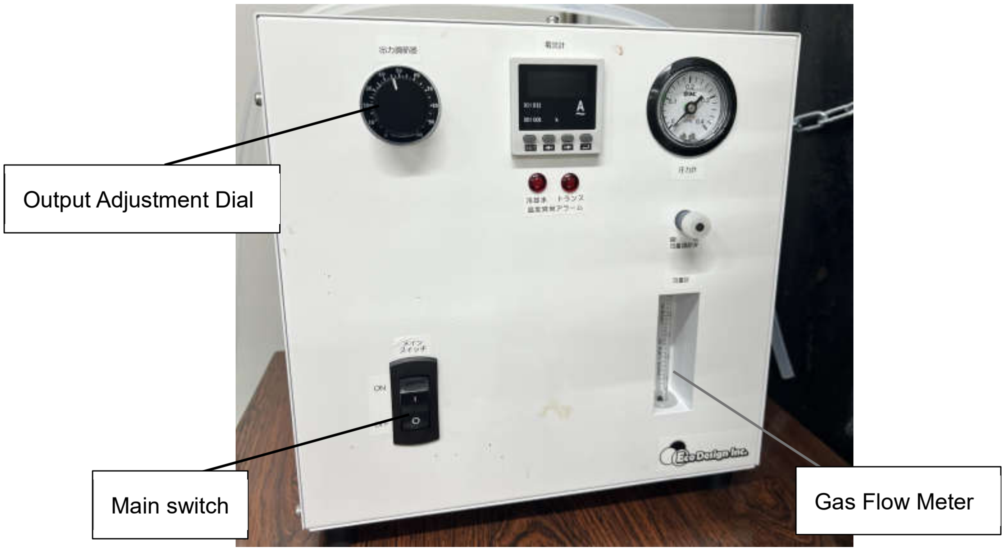

Figure S2 Diagram of ozone generator

The flow rate of oxygen gas containing ozone was measured with a gas flow meter at 1.0 L min^{-1}. Figure S2 shows the ozone gas generator. The relationship between the value indicated by the dial and the concentration of ozone gas generated at that time is shown in the figure S3.

Dial	Ozone conc (mg L^{-1})
0	0
10	6
20	15
30	27
40	37
50	44
60	50
70	56
80	62
90	65
100	67

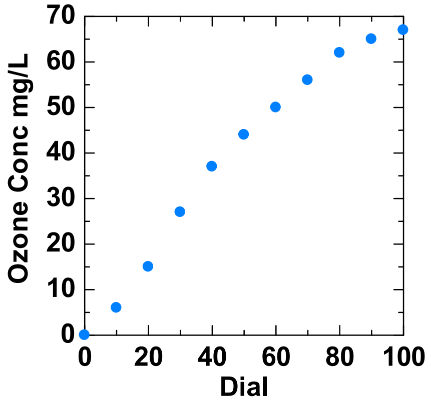

Figure S3 Relation of dials and ozone concentration

3. Analytical HPLC chromatograms

Table.S2 ccMA concentration and peak area

ccMA Conc.	Peak area	
mmol/L	257nm	277nm
0.992	11408628	6810495
0.496	5773772	3486501
0.298	3498612	2091344

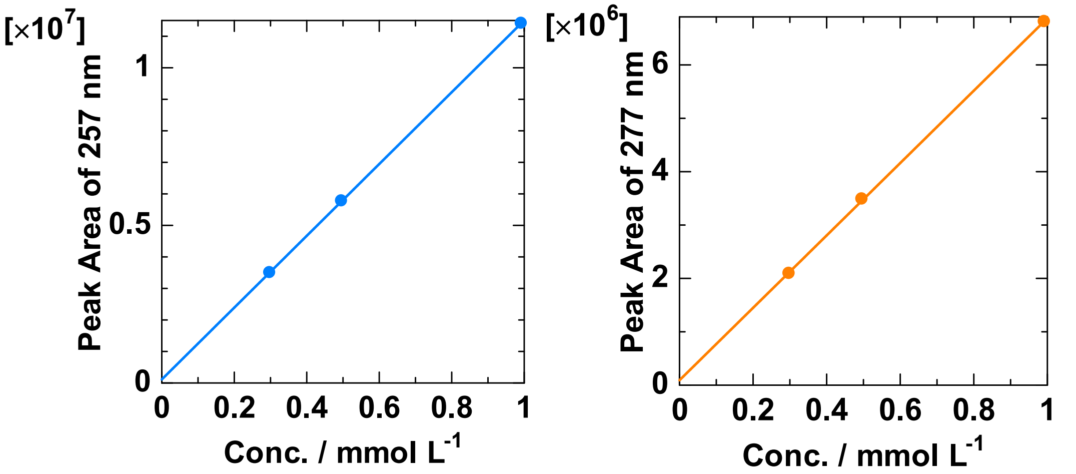

Area = 1.14×10^7 × Conc. + 1.13×10^5

R^2=0.999997

Area = 6.78×10^6 × Conc. + 9.25×10^4

R^2=0.99994

Figure S4 Calibration curve for ccMA

4. Conditions for HPLC Measurements

Waters Alliance 2695 separations module with PDA detector (Waters Alliance 2998) was used. Inert Sustain C18® 5 µm 14 × 150 mm (GL Science, Tokyo, Japan) was used as a separation column. The separation was performed in gradient mode using the following two solvents:

Liquid A: Add 1 mL of formic acid to 999 mL of ultrapure water.

Liquid B: Add 1 mL of formic acid to 999 mL of acetonitrile.

Table.S1 Conditions for HPLC Measurements

Time / min	Flow / mL/min	%A	%B
0	1.00	80.0	20.0
6.00		80.0	20.0
6.01		10.0	90.0
11.00		10.0	90.0
11.01		80.0	20.0
15.00		80.0	20.0

Determination of ccMA

The determination of ccMA synthesized was performed using the literature value (Sistrom, W. R.; Stanier, R. Y. *J. Biol. Chem.* **1954**, *210*, 821) of the molar absorption coefficient, ε = 17,300 (257 nm). The ε value of the final purified ccMA we synthesized was 17057 (257 nm), which was almost identical to the literature value.

5. Chromatograms

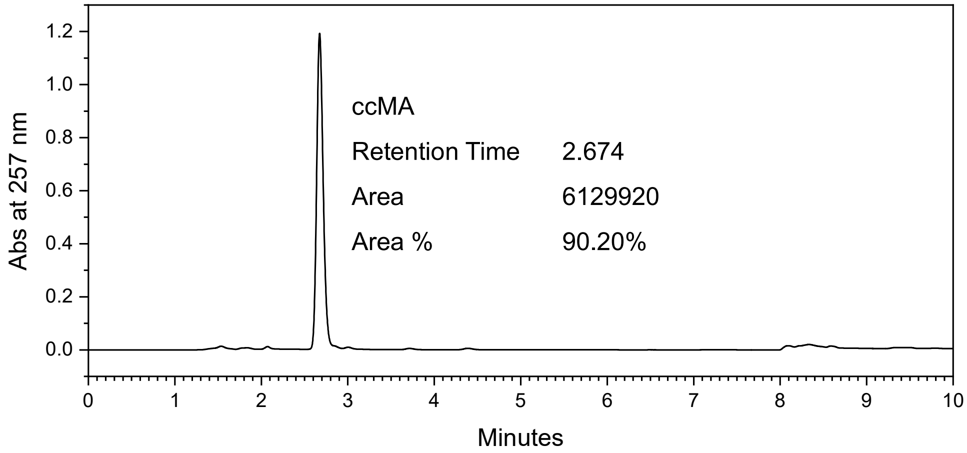

Figure S5 Chromatogram for Entry 5 in Table 5.

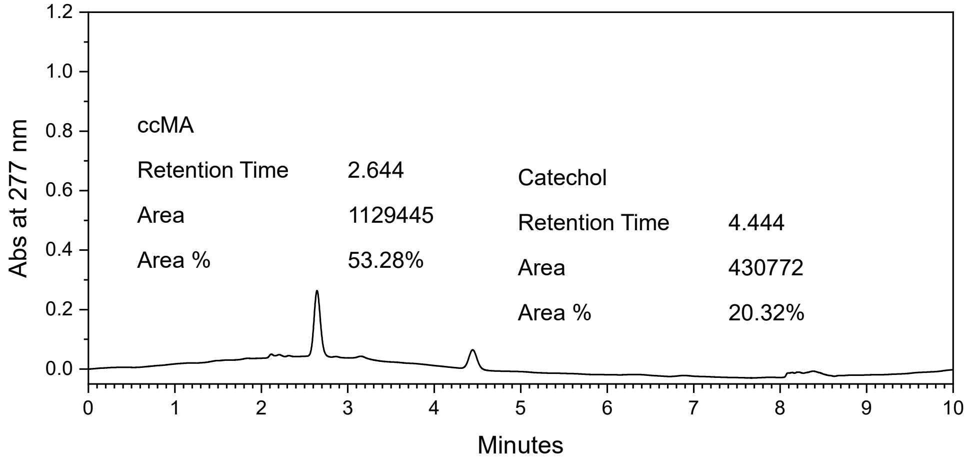

Figure S6 Chromatogram for Entry 2 in Table 2.

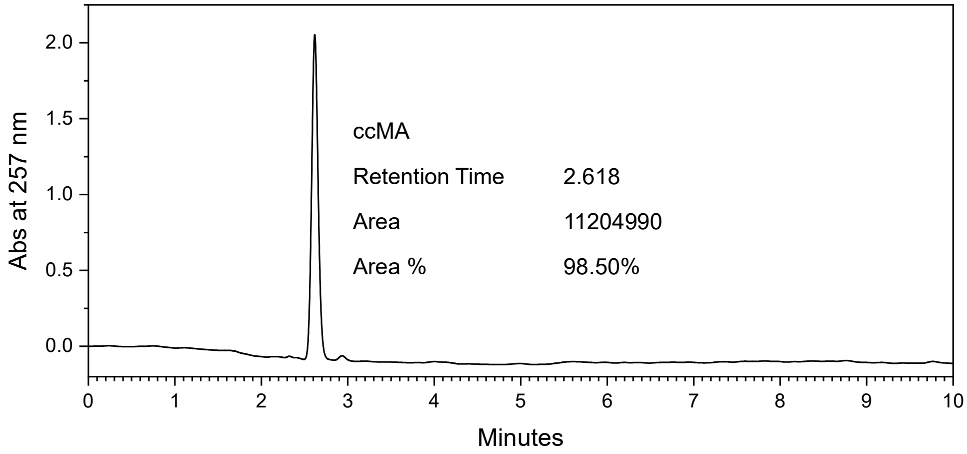

Figure S7 Chromatogram for Entry 2 in Table 3

6. Crystallization of ccMA

To the solution after the reaction, 200 mL of water was added to dissolve the suspension of ccMA. IPA was removed under reduced pressure using an evaporator to obtain an aqueous solution of the reactants. The pH of the aqueous solution was monitored with a pH meter and neutralized to pH 7 by adding HCl. A 40 g (20% w/v) of powdered activated carbon was added and stirred at room temperature all night. Centrifugation was performed at 4000 rpm for 10 minutes, and the supernatant was collected. The supernatant was cooled in an ice bath. The pH of the solution was monitored and adjusted to pH 2 by adding HCl. White powdery crystals were formed. The solution was cooled at 4 °C in a refrigerator overnight. Centrifugation was performed again at 4000 rpm for 10 minutes and ccMA was collected as precipitate. The recovered powdered crystals were vacuum dried at 45 °C for 6 hours to obtain white powdered crystals as shown in the figure S8.

Yield ... 4.57 g

Yield based on catechol (0.1 mol) ... 32%.

Purity ... 98.6%

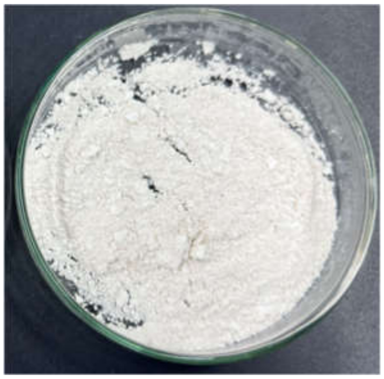

Figure S8 Crystal of ccMA

Ref. Li, Q.; Wang, D.; Wu, Y.; Li, W.; Zhang, Y.; Xing, J.; Su, Z. One Step Recovery of Succinic Acid from Fermentation Broths by Crystallization. *Sep. Purif. Technol.* **2010**, *72* (3), 294−300.

7. NMR spectra

^1H NMR (DMSO-d_6, 500 MHz) δ 12.71 (br s, 2H), 7.72 (d, 2H, J=8.3 Hz), 5.9-6.0 (m, 2H)

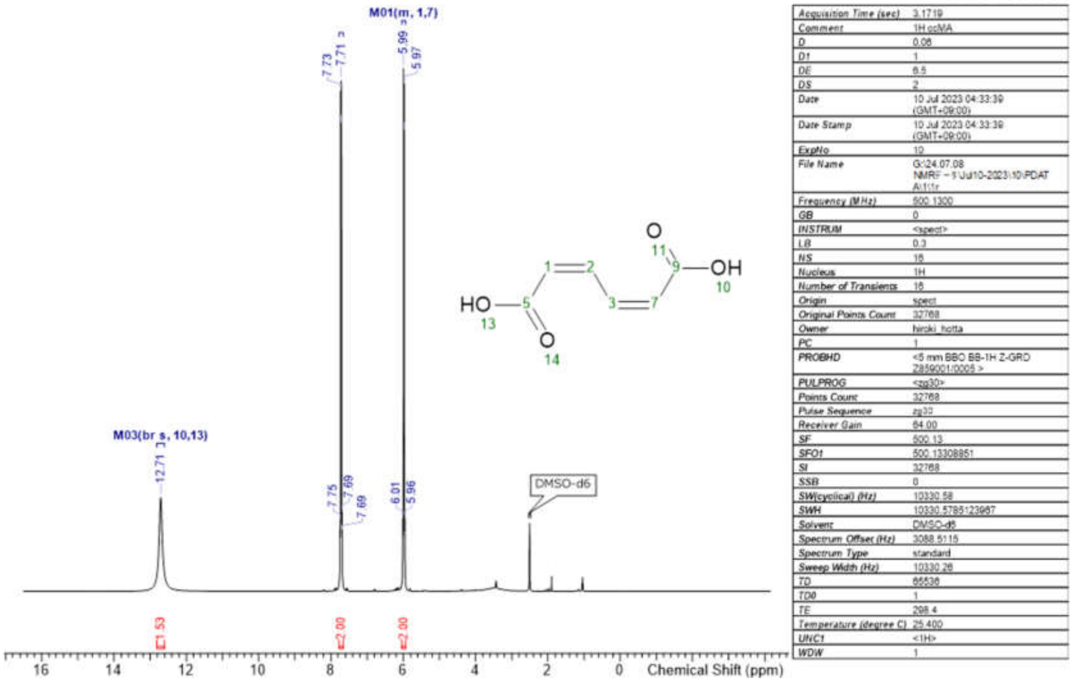

Acquisition Time (sec)	3.1719
Comment	1H ccMA
D	0.08
D1	1
DE	6.5
DS	2
Date	10 Jul 2023 04:33:39 (GMT+09:00)
Date Stamp	10 Jul 2023 04:33:39 (GMT+09:00)
ExpNo	10
File Name	G:\24.07.08 NMRF - 1\Jul10-2023\10\PDATA\1\1r
Frequency (MHz)	500.1300
GB	0
INSTRUM	<spect>
LB	0.3
NS	16
Nucleus	1H
Number of Transients	16
Origin	spect
Original Points Count	32768
Owner	hiroki_hotta
PC	1
PROBHD	<5 mm BBO BB-1H Z-GRD Z859001/0005 >
PULPROG	<zg30>
Points Count	32768
Pulse Sequence	zg30
Receiver Gain	64.00
SF	500.13
SFO1	500.13308851
SI	32768
SSB	0
SW(cyclical) (Hz)	10330.58
SWH	10330.5785123967
Solvent	DMSO-d6
Spectrum Offset (Hz)	3088.5115
Spectrum Type	standard
Sweep Width (Hz)	10330.26
TD	65536
TD0	1
TE	298.4
Temperature (degree C)	25.400
UNC1	<1H>
WDW	1

Figure S9 ^1H NMR of ccMA
